# Dysregulated Transcriptional Control in Prostate Cancer

**DOI:** 10.3390/ijms20122883

**Published:** 2019-06-13

**Authors:** Simon J. Baumgart, Ekaterina Nevedomskaya, Bernard Haendler

**Affiliations:** Research & Development, Pharmaceuticals, Bayer AG, Müllerstr. 178, 13353 Berlin, Germany; simon.baumgart@bayer.com (S.J.B.); ekaterina.nevedomskaya@bayer.com (E.N.)

**Keywords:** prostate cancer, gene transcription, single-nucleotide polymorphism, super-enhancer, enhancer RNA

## Abstract

Recent advances in whole-genome and transcriptome sequencing of prostate cancer at different stages indicate that a large number of mutations found in tumors are present in non-protein coding regions of the genome and lead to dysregulated gene expression. Single nucleotide variations and small mutations affecting the recruitment of transcription factor complexes to DNA regulatory elements are observed in an increasing number of cases. Genomic rearrangements may position coding regions under the novel control of regulatory elements, as exemplified by the *TMPRSS2-ERG* fusion and the amplified enhancer identified upstream of the *androgen receptor* (*AR*) gene. Super-enhancers are increasingly found to play important roles in aberrant oncogenic transcription. Several players involved in these processes are currently being evaluated as drug targets and may represent new vulnerabilities that can be exploited for prostate cancer treatment. They include factors involved in enhancer and super-enhancer function such as bromodomain proteins and cyclin-dependent kinases. In addition, non-coding RNAs with an important gene regulatory role are being explored. The rapid progress made in understanding the influence of the non-coding part of the genome and of transcription dysregulation in prostate cancer could pave the way for the identification of novel treatment paradigms for the benefit of patients.

## 1. Introduction

The incidence of prostate cancer is the second highest in men and represents a leading cause of cancer mortality worldwide [1]. The recent progress made in whole-genome and transcriptome sequencing of primary, advanced, and metastasized tumor samples allowed major advances in the characterization of this heterogeneous disease [2,3,4,5,6]. Prostate cancer has a relatively low mutational burden of about one mutation per megabase [5,7], but multiple recurrent chromosomal losses and gains are frequent [2]. Gene fusions involving *E26 transformation-specific* (*ETS*) family members and coding mutations mainly affecting speckle-type POZ protein (SPOP), forkhead box protein A1 (FOXA1), and isocitrate dehydrogenase 1 (IDH1) are found in primary prostate cancer [2,3,4,5,8,9]. Several alterations affecting the androgen receptor (AR) and androgen signaling emerge in castration-resistant prostate cancer (CRPC) as an adaptation to treatment [2,3,4,5,8,10]. Late-stage, neuroendrocrine prostate cancer (NEPC) is characterized by the expression of transdifferentiation markers and the increased activity of oncogenes [2,11,12]. In addition, the previously unsuspected frequency of small non-coding alterations and the gene regulatory role of non-coding RNAs have recently been evidenced [4,13,14,15].

Here we review recent findings on dysregulated transcriptional control driven by genetic and epigenetic alterations and its contribution to oncogenic processes and resistance mechanisms in prostate cancer. We furthermore discuss how these mechanisms could be targeted to allow novel therapeutic options.

## 2. Promoters, Enhancers and Super-Enhancers

### 2.1. General Aspects

Tight spatiotemporal regulation of gene transcription is essential for normal development and function of all living cells. This is controlled by multiple regulatory units mostly positioned within 50 kilobase-pairs of the gene they regulate, but in some cases also megabase-pairs away [16,17]. Long-range connections between distant regulatory elements are established by looping [18,19].

**Figure 1:**
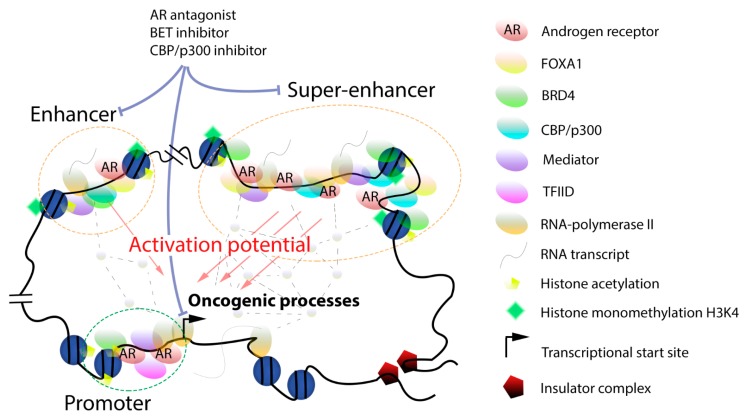
Interaction model for factors associated with enhancer and super-enhancer function in prostate cancer. The transcription activation potential of enhancers and super-enhancers is correlated to the levels of their associated factors. Transcription regulatory elements physically interact with their target genes and enable the local formation of a dense network of transcription factors (dotted lines with grey nodes), ultimately leading to sustained transcription by RNA Pol II. Compounds such as AR antagonists and BET or CBP/p300 inhibitors can block factors associated with enhancer or super-enhancer function.

The gene promoter located immediately upstream of the transcription start site directs the initial assembly of the transcriptional apparatus [20,21]. The proximal promoter element is usually rich in CpG islands situated upstream of the transcriptional start site and is characterized by high H3K4 trimethylation. It is responsible for the initial formation of a basal pre-initiation transcription complex including RNA polymerase II (Pol II), transcription factor II D and more than 40 other proteins (Figure 1) [20,21]. Following transcription initiation, the complex pauses and the transition to productive elongation necessitate the presence of the positive transcription elongation factor b (P-TEFb) complex made of cyclin-dependent kinase 9 (CDK9) and cyclin T [22]. This is controlled by cis-regulatory enhancer regions that interact with the promoter through the mediator, cohesin, the CCCTC-binding factor (CTCF) and Yin Yang 1 (YY1) complexes upon formation of chromosome loop structures [23,24].

Full, sustained gene transcription necessitates the interplay between the promoter and enhancer regions which are involved in both the recruitment of the basal transcription machinery and in post-recruitment steps (Figure 1) [25,26]. Active enhancers were originally found near or partially overlapping with promoter regions and have a length of a few hundreds of base-pairs [27]. They are located in open chromatin regions characterized by H3K27 acetylation and H3K4 monomethylation and by the presence of histone variants such as H3.3 and H2A.Z [28]. They usually do not contain CpG islands but have binding motifs for important regulators of the CCAAT-enhancer-binding proteins (C/EBP) which interact in a cooperative fashion with numerous proteins involved in gene transcription [29]. Additional enhancer hallmarks include H3K79 trimethylation and CREB-binding protein (CBP)/p300 recruitment [30,31]. Enhancers are bound by the bromodomain and extra-terminal (BET) protein BRD4 and by members of the mediator and P-TEFb complexes [32,33,34,35]. Recent single-cell analysis of gene transcription revealed further details on the inter-dependencies of these factors in enhancer function [36]. Bidirectional enhancer transcription driven by RNA Pol II and leading to the synthesis of non-coding enhancer RNAs (eRNAs) has been evidenced [25,37]. These eRNAs promote transcription by facilitating the formation of DNA loops between regulatory regions, which ultimately leads to highly increased, productive transcriptional activity ending up with the eviction of RNA Pol II at the 3′-tail of transcribed genes. Recent studies mainly based on chromatin conformation techniques have blurred the distinction between enhancers and promoters, as a number of promoters can also act as enhancers for the long-range regulation of distant genes and also engage in interactions with other promoters [38]. Long-range, inter-nucleosomal contacts controlled by the N-terminal tails of histones are essential and possibly rate-limiting for the cooperation between enhancers and promoters [39].

With the advent of technologies for analysis of protein-DNA interaction and of chromosomal conformation, genome wide landscapes of regulatory regions have been unraveled and the impact of the chromosomal architecture on gene expression and disease revealed [40,41,42]. This allowed the identification of a novel category of clustered enhancers dubbed super-enhancers, with an essential role in cell-specific gene expression and identity (Figure 1) [43,44,45,46,47,48,49]. Genes regulated by super-enhancers are typically expressed at higher levels when compared to enhancer-controlled genes. Importantly, super-enhancers are found in transient biomolecular condensates, which also include gene promoters, transcription factors, coactivators, and RNA Pol II, whose role is to increase the local concentration of these complexes around defined gene regions looped into them and to boost transcription [50,51,52]. The formation of phase-separated droplet compartments improves transcription fidelity, which is essential to maintain cellular phenotype [50,51,52]. On the other hand, super-enhancers are much more susceptible to changes in levels of bound transcription factors or coactivators, which further underlines the importance of local cooperative interactions for their transcriptional output [44,47,50]. Super-enhancers typically span tens of thousands of base pairs and are densely bound by lineage-specific transcription factors, by members of the mediator multi-subunit complex, and by BRD4 which engage in short-term lived interactions via intrinsically disordered regions [51]. The transcription factors Yes-associated protein (YAP) and transcriptional coactivator with PDZ-binding motif (TAZ) are important BRD4-interacting binders at super-enhancers which stimulate the expression of numerous genes involved in cell proliferation [53]. The P-TEFb complex is also localized at super-enhancers [54,55]. Cyclin-dependent kinase 7 (CDK7) belongs to the transcription factor II H complex and controls RNA Pol II activity by phosphorylating its C-terminal tail, thereby acting as a master regulator of super-enhancer activity [56]. An essential role of CDK7 in controlling super-enhancer-driven oncogenes in different tumor types has been evidenced using various inhibitors [57,58,59,60,61]. Cyclin-dependent kinase 12 (CDK12) and cyclin-dependent kinase 13 (CDK13) regulate transcription elongation and the expression of super-enhancer-dependent transcription factor genes [62]. Super-enhancers are usually flanked by boundary elements representing binding sites for CTCF that prevent expression of neighboring genes [24]. Recent progress made in three-dimensional chromatin capture techniques led to the definition of topologically associating domains (TADs) which specify chromatin compartments delimitated by distinctive elements [63]. TADs are dynamic structures formed by the cohesin complex and flanked by CTCF [63,64]. They are found in chromatin regions with very high levels of H3K27 acetylation and H3K4 monomethylation. Strong TAD boundaries are associated with high CTCF levels and frequently insulate super-enhancers [65]. Non-coding eRNAs facilitating the interactions with promoter regions to regulate downstream gene expression [37] and that may influence the tissue- and cell-specific activity of super-enhancers have been identified [66,67]. Algorithms to identify super-enhancers such as ROSE and archiving databases such as dbCORC, and SEdb are now available [45,68,69]. Recently, a sub-classification between non-hierarchical and hierarchical hub and non-hub super-enhancers has been proposed, the latter being more frequently associated with disease risks [70].

### 2.2. Non-Coding Cancer Driver Mutations

**Figure 2:**
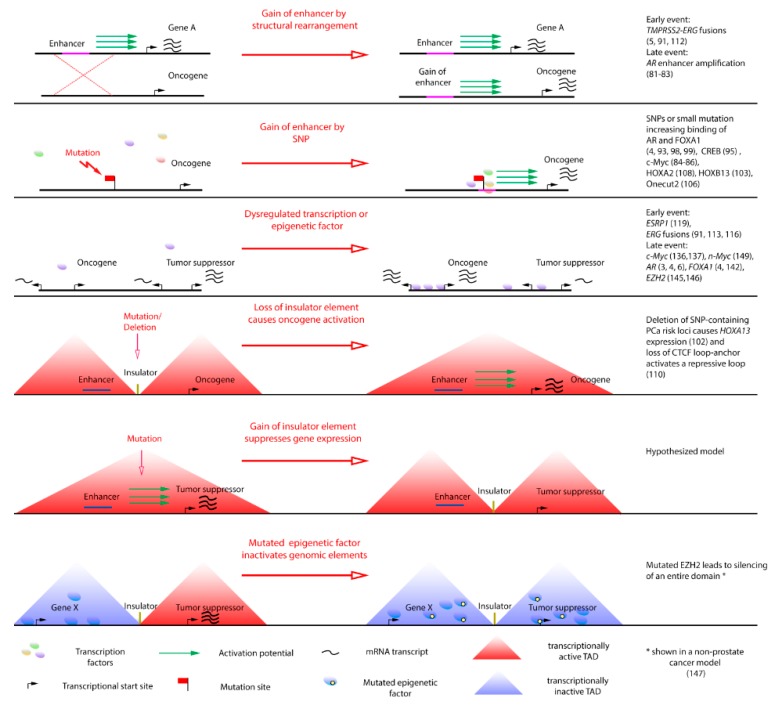
Events disrupting gene regulatory elements or TADs lead to oncogenic transcriptional dysregulation. The normal cell state (left side) is dysregulated by an oncogenic event which leads to a malignant cell state (right side). TADs comprise insulated genomic regions which can be transcriptionally active (red triangles) or inactive (blue triangles). Examples and selected references for each event are given in the right-hand column, with a focus on prostate cancer.

With the recent advances in genomic sequencing, more and more small-scale driver events such as point mutations and small insertions have been identified (Figure 2). One of the first examples is the discovery that about 5% of T cell acute lymphoblastic leukemia cases harbor small DNA insertions that create novel binding sites for recruitment of myeloblastosis (MYB) transcription factors and of the histone acetyltransferase CBP upstream of the T-cell acute lymphocytic leukemia protein 1 (TAL1) oncogene, thus creating a strong tumor-driving super-enhancer [71]. Another example is the finding of point mutations in the promoter of the *telomerase reverse transcriptase* (*TERT*) gene that create novel transcription-factor binding sites. These mutations were initially found in melanoma but later also in several other tumor types [72,73,74]. The *TERT* gene is usually silenced in differentiated cells and the identified promoter mutations lead to increased expression and telomerase reactivation and eventually to uncontrolled cell proliferation.

Non-coding driver alterations that involve large genomic rearrangements such as chromosomal translocations, focal amplifications, deletions, and viral insertions have been described for a long time in cancer and the recent advances made in genome-wide analyses and chromatin conformation techniques have led to a better understanding of these events (Figure 2). These changes may for instance position an oncogene under the control of a strong promoter or enhancer or lead to silencing of a tumor suppressor gene [44,75,76]. Also, changes affecting TAD boundary regions leading to the formation of new chromatin loops and dysregulated gene transcription have been described [77]. Aberrant enhancer and super-enhancer activity plays a role in different tumor types. Super-enhancer regions and strong TAD boundaries are often co-duplicated in tumors [65]. Also, a pan-cancer analysis of somatic copy number alterations in non-coding genomic regions led to the identification of six super-enhancers controlling the expression of four genes in different tumors [78]. Two focally amplified super-enhancers are responsible for *c-Myc* overexpression in lung and endometrial tumors [78]. Other studies report on super-enhancers aberrantly active in ependymomas [79] or rearranged in breast cancer [80].

Examples of SNPs and aberrant enhancer and super-enhancer activity leading to transcription dysregulation in prostate cancer, for instance due to aberrant expression of the *AR* or of the *c-Myc* gene [81,82,83,84,85,86], are detailed below.

## 3. Dysregulated Transcription Control in Prostate Cancer

Dysregulation gene expression is observed both in early and late-stage prostate cancer. Important examples are detailed below and summarized in Figure 2.

### 3.1. Early Events

Inherited genetic markers account for more than half of prostate cancer risk factors and include coding and non-coding variants [13,87]. In total, 50–100 single nucleotide polymorphisms (SNPs) have been linked to prostate cancer development [88,89] and validation studies indicate that many of them have a regulatory function and control gene expression [88]. Another study used chromatin conformation analysis to identify prostate cancer risk-associated SNPs and demonstrated the role of CTCF-binding motifs and three-dimensional chromatin folding in preventing enhancer function from spreading towards neighboring gene regions [18]. Importantly, comparison of prostate cancer and normal samples reveals that AR binding is redistributed in tumors compared to healthy tissue samples, leading to important transcriptome changes [90]. In addition, a colocalization of FOXA1 and homeobox protein B13 (HOXB13) at sites with elevated AR binding in tumors is observed. Large genetic changes such as gene amplifications and deletions are also frequently reported in early tumors [5,91] and have been recently reviewed [6,92].

#### 3.1.1. Regulatory SNPs

A genome wide association study (GWAS) was used to characterize 77 prostate cancer risk loci and find functional SNPs [93]. Many of these SNPs localize at putative enhancers with high H3K27 acetylation levels. Further analysis for functionality revealed that in several cases the binding of transcription factors such as the AR, FOXA1, and NK3 homeobox 1 (NKX3-1) was affected by the sequence variations [93].

The SNP rs10993994 leads to reduced expression of the gene coding for *prostate secretory protein 94* (*PSP94*) and represents a causal variant for prostate cancer risk [94]. This was linked to differential recognition of the SNP by cAMP response element-binding protein (CREB) [95]. PSP94 is a major prostate secretory protein which interacts with cysteine-rich secretory proteins [96] and is deemed to be a tumor suppressor due to its role in apoptosis [97].

Multiple variants were found in an enhancer region that loops to the *Sry-related HMG box-containing 9* (*SOX9*) gene [98]. Two SNPs, rs8072254 and rs1859961, that affect binding by the AR or by FOXA1 and activating protein-1 (AP-1), respectively, have been identified in this region.

Allele-specific enhancer activity was also shown for the prostate cancer risk SNPs rs2659051, rs10936845, rs9925556, rs6057110, and rs2742624 [99]. The impact of the polymorphisms on recognition by the AR, FOXA1, HOXB13 and GATA-binding factor 2 (GATA2), and on H3K27 acetylation was described.

A variant that may predispose to prostate cancer was reported at 7p14.3 [100]. It is modulated by the AR and C/EBP and correlates with mutations in the gene encoding SPOP.

Several variants of the 8q24 region linked to variable increases of prostate cancer risk, depending on the ethnic origin, have been reported [101]. This region harbors a functional enhancer and may alter *c-Myc* gene expression, which promotes tumor growth [84,85,86,88]. Indeed, long-range interacting loops were identified between the *c-Myc* region and a functional enhancer located in this region in prostate cancer cell lines by using chromosome conformation capture techniques [84,85,86].

Several SNPs in the 7p15.2 locus are correlated with increased prostate cancer susceptibility and this was experimentally tested by deleting the region. This led to the identification of a repressive long-range loop spanning over 800 kilobase-pairs that controls *HOXA13* expression [102].

The SNP rs339331 identified in intron 4 of the *regulatory factor X6* (*RFX6*) gene enhances binding of the homeobox family member HOXB13, thus leading to transcription upregulation [103]. Interestingly, *HOXB13* is itself linked to hereditary prostate cancer, due to a number of coding mutations [104]. Recent data show that HOXB13 forms a heterodimer with the AR V7 splice variant, which is associated with therapy resistance to drive specific gene expression programs [105].

The SNP rs7463708 is responsible for elevated binding of ONECUT2, an AR-interacting transcription factor, to an enhancer region governing expression of the non-coding *RNA prostate cancer-associated transcript 1* (*PCAT1*) [106]. Importantly, ONECUT2 is a master regulator of AR signaling and a survival factor in metastatic CRPC [107]. In addition, preclinical models show that *PCAT1* overexpression is able to stimulate cancer growth.

The SNP rs11672691 is linked to prostate cancer predisposition and aggressiveness. It is located in an intron of the non-coding RNA *prostate cancer-associated transcript 19* (*PCAT19*) which possesses enhancer-like features. It creates a novel binding site for the homeobox family member HOXA2, leading to increased expression of *PCAT19* and of the *cell adhesion molecule carcinoembryonic antigen-related cell adhesion molecule 21* (*CEACAM21*) gene [108]. *HOXA2* is overexpressed in prostate cancer and its silencing reduces tumor proliferation in vitro and in vivo. A separate analysis of the same SNP region indicates that it is associated with the expression of different PCAT19 isoforms, depending on the exact DNA sequence and recognition by the NKX3-1 and YY1 transcription factors [109]. Both a promoter and an enhancer function are reported for this risk SNP region [109].

SNPs associated with CTCF sites are linked to prostate cancer risk [110]. These sites are involved in long-range chromatin loops and their deletion dramatically affects the expression of genes located between them.

The expanding use of GWAS and ongoing discovery of novel SNPs thanks to deeper sequencing of tumor DNA increase the need for rapid functional assessments. Genome-editing technologies such as clustered regularly-interspaced short palindromic repeats with Cas9 nuclease (CRISPR-Cas9) have greatly helped to dissect the impact of SNPs on different diseases, including cancer [111]. Also, translation of these findings into the clinic for diagnostic and potential therapeutic use will necessitate dedicated technologies.

#### 3.1.2. *ERG* Translocation

Analysis of primary prostate cancer samples shows that translocation events leading to fusions of an *ETS* family member to an AR-driven promoter are found in about half of the patients [5,91,112]. A recent comprehensive study of chromatin modification and transcription factor binding allowed to classify primary prostate cancer with *ETS-related gene* (*ERG*) translocation into clusters characterized by high or low ERG expression or by a NEPC-like profile [91]. The most frequent translocation positions the *ERG* coding region downstream of the androgen-dependent *transmembrane protease serine 2* (*TMPRSS2*) gene promoter due to the deletion of an intervening region and conducts to increased expression of the ERG transcription factor [113]. Moreover, elevated ERG expression itself is associated with large changes in chromatin organization [114]. It leads to recruitment of transcription factors with an essential role in prostate function and to transcription from novel cis-regulatory elements [113,115]. This is linked to the appearance of attending super-enhancers with high H3K27 acetylation levels, including one located in the fused *TMPRSS2* promoter and potentially responsible for *ERG* overexpression [113]. ERG and AR-associated long-range chromatin loops leading to coordinated regulation of downstream target genes have been described [116]. Another report shows that ERG interacts with BRD4 and colocalizes at numerous target genes that are essential for cell proliferation and invasion [117]. A recently identified downstream target of ERG is CBP/p300-interacting transactivator 2 (CITED2), a molecular chaperone that promotes the translocation of p300 and protein arginine N-methyltransferase 5 (PRMT5) and the formation of a multimeric complex with nucleolin which stimulates cell migration, ultimately leading to prostate cancer metastasis [118].

#### 3.1.3. *Epithelial Splicing Regulating Protein 1* (*ESRP1*) Gene Duplication

A comprehensive study in early-onset prostate cancer patients combining whole genome sequencing, epigenetic marks, and transcriptome analyses allowed the identification of recurrent duplications of the gene encoding *ESRP1* [119]. These duplications lead to elevated ESRP1 expression and correlate with disease aggressiveness. This finding additionally underlines the role of aberrant splicing in prostate cancer [120].

#### 3.1.4. *Phosphatase and Tensin Homolog* (*PTEN*) Inactivation

*PTEN* deletion is observed in about 20% of primary prostate cancer samples beside DNA methylation leading to decreased expression [121] and mutations that inactivate the protein [122]. This occurs via several mechanisms including deletion at 10q23 [123], ultimately suppressing an essential brake for phosphoinositide 3-kinase (PI3K) signaling and representing a key driver in proliferation as outlined in many reports [124,125].

### 3.2. Advanced Prostate Cancer

Several large genomic alterations including *AR* gene amplification and *PTEN* loss which are already seen in primary tumors are observed at a higher frequency in metastatic samples [3,4,6]. Concerning non-coding regions, a search for focal amplifications in different tumor types, including prostate cancer, allowed the identification of super-enhancers and their putatively regulated target genes [46]. The role of the histone variant H2A.Z in activating novel enhancers exemplifies the impact of enhancer gain on disease outcome worsening [126]. Details on key genetic events directly affecting the transcription of genes with an essential role in prostate cancer are outlined below.

#### 3.2.1. Binding of BRD4 and Interacting Proteins at Gene Regulatory Elements

BRD4 is a global reader of the activating histone acetylation mark [127,128]. It binds mainly to enhancers and super-enhancers of numerous genes involved in cell proliferation and is involved in tumor cell transcriptional addiction [49,53,129]. A preferential binding of BRD4 to many SNPs located in enhancers and associated with prostate cancer risk has been reported [129]. Further refinement is achieved by incorporating the binding profiles of mediator complex proteins and H3K27 acetylation marks, which are all characteristic features of super-enhancers [129]. Importantly, BRD4 and other bromodomain and extra-terminal (BET) proteins regulate AR signaling at different levels. They control *AR* expression by binding to acetylated chromatin regions found at different locations in the *AR* gene body [130]. In addition BRD4 forms a complex with the AR and both proteins colocalize to regulatory regions of several androgen target genes, including the one coding for prostate-specific antigen (PSA) [131]. Interestingly, BRD4 binding is more powerful than AR binding to super-enhancers for identification of risk loci linked to prostate cancer [129]. BRD4 associates with the P-TEFb complex which releases paused RNA Pol II [132]. The P-TEFb complex contains CDK9 which phosphorylates the AR [22] and can be activated by androgen-regulated eRNAs [133], thus further sustaining signaling. In addition, BRD4 also interacts with ERG to control common genes upregulated in CRPC [117]. Another protein that binds to BRD4 and to the AR is YAP, a transcription factor found at super-enhancers [53,134]. YAP is involved in the transition to androgen-independent, AR-mediated transcription ultimately leading to castration resistance [135]. Another important downstream target of BRD4 is *c-Myc*, an oncogene which is amplified in about 30% of late-stage prostate tumors. Importantly, the expression of *AR* and *c-Myc* correlates during progression to metastatic CRPC and c-Myc regulates *AR* expression [136], suggesting an essential role for both factors [137]. Finally, the regulatory role of BET proteins for an enhancer that controls glucocorticoid receptor expression, which is de-repressed in advanced tumors, has been reported [138].

#### 3.2.2. Acquired *AR* Enhancer and Androgen-Dependent Neo-Enhancers

Amplification and overexpression of the *AR* gene are by far the most frequent resistance mechanisms observed in prostate cancer patients treated with drugs that suppress androgen signaling. This allows the restoration of the AR pathway despite castrate levels of circulating male hormones. Recently, a genomic rearrangement leading to tandem duplication of an intergenic enhancer element located 600–700 kilobase-pairs upstream of the *AR* gene has been identified in a large subset of metastatic CRPC patients [81,82,83]. Amplification of this enhancer often parallels that of the *AR* gene and both changes concur to maintain high androgen signaling in advanced tumors. The functionality of this *AR* enhancer region is supported by the facts that it is looping to the *AR* gene and located in an open, highly acetylated chromatin environment in CRPC but not in localized tumor samples, and experimentally by silencing and knock-in experiments in a prostate cancer cell line [83]. Another study shows that nucleosomes containing acetylated H2A.Z are incorporated at enhancers associated with AR activity and contribute to the formation of neo-enhancers in prostate cancer [126]. These neo-enhancers have typical characteristics of active enhancers including H3K27 acetylation and increased eRNA transcription [126].

Additional research should provide further information on how enhancers around the *AR* gene sustain constant, high *AR* expression and on the impact of neo-enhancers on downstream androgen signaling. Technologies that capture the looping of chromatin and show enhancer-promoter association will pave the way for new insights in this area and possibly also lead to the identification of novel resistance mechanisms.

#### 3.2.3. *Nuclear Enriched Abundant Transcript 1* (*NEAT1*) and *FOXA1* Promoter Mutations

Analysis of whole genome sequencing data allowed the identification of non-coding driver mutations in metastatic prostate cancer [4]. Mutations in the gene for the long non-coding RNA *NEAT1* were significantly enriched in metastatic tumors treated by androgen deprivation therapy, compared to primary tumors. Mutations were also found in the promoter of the *FOXA1* gene which codes for a transcription factor that modulates AR binding locations [4].

#### 3.2.4. Reprogramming to Neuroendocrine Phenotype

The advent and earlier use of effective AR-targeting agents has led to the increased appearance of resistance mechanisms where prostate tumor cells acquire novel neuroendocrine features and become independent of the AR signaling axis [139]. This is accompanied by a loss of *PSMA* expression [140], which is often used for detection and staging of prostate cancer [141]. The pioneer factor FOXA1 is frequently expressed in NEPC and may represent a useful progression marker [142]. Expression of *SAM pointed domain-containing ETS transcription factor* (*SPDEF*) belonging to the *ETS* transcription factor gene family is lost upon androgen deprivation therapy and may contribute to the development of NEPC [143]. NEPC is highly aggressive and has undergone extensive reprogramming involving epigenetic players and long, non-coding RNAs [144]. Increased expression of the H3K27 histone methylase *enhancer of zeste homolog* (*EZH2*) gene is a common feature of advanced prostate cancer and NEPC and is observed both in patients and in tumor models [145,146]. EZH2 mutations leading to silencing of TADs and tumor suppressor genes located within these domains due to changes in H3K27 trimethylation have recently been described [147]. Importantly, androgen deprivation leads to increased EZH2 activity and ultimately promotes angiogenesis, which is elevated in NEPC [148]. The transcription factor *N-Myc* is overexpressed in NEPC and this is sufficient to induce transformation and androgen independence [149]. Experimentally, high N-Myc levels lead to shift of different prostate cancer models and of PTEN-negative mouse organoids towards androgen independence by strengthening the EZH2 and AKT signaling axis [149]. Essential roles of the repressor element 1-silencing transcription factor (REST) and its downstream repressed target, the long non-coding RNA *HOTAIR*, have recently been evidenced [150,151,152]. *REST* expression is controlled by the RNA splicing factor serine/arginine repetitive matrix 4 (SRRM4), a main driver of NEPC progression [153]. Importantly, *SRRM4* expression in castration-resistant prostate cancer correlates with poor patient survival [154]. A comprehensive analysis of the changes in long non-coding RNA expression found in NEPC that take place upon androgen deprivation treatment revealed several candidates involved in this process [155]. As whole-transcriptome data from NEPC patient samples are now available [2,11,12,142,153,155,156] a detailed understanding of the mechanisms underlying dysregulated gene expression during this disease stage should be unveiled soon.

## 4. Targeting Dysregulated Gene Transcription

Androgen signaling is essential for maintenance of normal prostate development and function. It is also a main driver of prostate cancer development due to reprogramming leading to a redistribution of AR binding sites and large transcriptome changes [90,157]. This is accompanied by a relocalization of FOXA1 and HOXB13 and the formation of novel genomic AR subcomplexes [19]. Importantly, transduction with these two pioneer transcription factors is sufficient for reprogramming normal prostate epithelium into transformed tumor cells [90]. The AR pathway has been successfully addressed in the treatment of early and late-stage prostate cancer for many years, originally with surgical or chemical castration and later with AR antagonists and androgen synthesis inhibitors (Figure 1) [158,159,160,161,162]. The recent identification of an enhancer hijacked by the *AR* gene to upregulate expression adds a novel facet to the strategies used by tumors to sustain hormone action and overcome androgen deprivation. Previously described mechanisms leading to restoration of androgen signaling include epigenetic *AR* gene activation linked to DNA demethylation and histone modifications [163,164,165], AR mutations [166], generation of AR splice variants [167], and increased coactivator function [168]. This vindicates the identification and development of novel AR-targeting compounds with improved properties such as the next-generation AR antagonists enzalutamide, apalutamide and darolutamide [158,159,161,169,170,171,172,173]. A dual inhibitor of AR function and CYP17A1 lyase activity is currently in the clinical dose escalation phase [174]. Less advanced compounds include AR degraders [175,176,177] and agents addressing AR splice variants deprived of the ligand-binding domain [178]. Attempts to reduce AR levels using a specific antisense nucleotide have been evaluated clinically but with little success [159,179]. In another approach, AR transcript and protein levels could be reduced by targeting deubiquitinases, and first in vitro efficacy data based on this approach have been reported [180].

Translocations leading to *ERG* overexpression are observed in a large group of primary prostate cancer cases and are maintained in CRPC. Efforts to identify compounds that interfere with ERG function have been reported. The described inhibitors act indirectly and show anti-tumor efficacy in different prostate cancer models [181,182].

BRD4 is an essential component of super-enhancers which binds to acetylated histones and possibly to other acetylated proteins via its two bromodomains. Inhibitors targeting the bromodomains of BRD4 and related BET proteins show efficacy in preclinical prostate cancer models (Figure 1) [130,161,183]. Several compounds were advanced to the clinic and a few trials focusing on prostate cancer patients are currently ongoing [184].

The P-TEFb complex contains CDK9, which has been successfully targeted to inhibit preclinical prostate cancer models in vitro and in vivo [24]. First compounds that blockCDK9 have entered the clinic, but in many cases, lack of specificity leading to off-target side-effects has limited their application [22]. Highly selective CDK9 inhibitors have recently been described and may have a better therapeutic window [185,186].

CDK7 is another kinase involved in super-enhancer function. The availability of a potent and specific inhibitor has much helped in understanding the impact of super-enhancers in different tumor types [44], however, no data for prostate cancer are currently available.

The related CBP/p300 histone acetyltransferases are essential mediators of different acetylation marks including H3K27 acetylation [187] and also important AR coactivators linked to prostate cancer progression [188,189]. Selective inhibitors addressing either the bromodomain or the enzymatic activity have been described recently and anti-proliferative efficacy reported in vitro and in vivo in several preclinical prostate cancer models (Figure 1) [190,191,192,193]. The CBP/p300 bromodomain inhibitor CCS1477 has recently entered a clinical phase I study focusing on prostate cancer [192].

The involvement of EZH2 as a master epigenetic player in late-stage tumors and in NEPC is documented by numerous studies [145,148]. Importantly, EZH2 directly stimulates *AR* expression, independently of its methyltransferase activity, by binding to the *AR* gene promoter [194]. EZH2 inhibition leads to apoptosis when combined with chemotherapeutic agents [195] and is efficacious in docetaxel-resistant tumor cells [196]. Significant in vivo efficacy was reported when combining an EZH2 inhibitor with enzalutamide for treatment of a prostate cancer xenograft model [194]. Several potent inhibitors targeting EZH2 have been identified in the last years and tested in numerous preclinical tumor models [197]. A clinical study combining an EZH2 inhibitor with the AR antagonist enzalutamide or the CYP17A1 inhibitor abiraterone acetate has very recently been initiated in metastatic CRPC patients [198]. EZH2 interacts with the embryonic ectoderm development (EED) and SUZ12 proteins to form the polycomb repressive complex 2 (PRC2) [199]. Several inhibitors of EED that bind to the H3K27me3 pocket and block PRC2 methyltransferase activity have been reported [200,201]. One of them, MAK683, is being evaluated in lymphoma and in solid tumors, including prostate cancer.

N-Myc and the Akt signaling pathways are important players in NEPC [149]. N-Myc is stabilized by the mitotic kinase Aurora A and a specific inhibitor was evaluated in NEPC, but the primary endpoint was not met despite the fact that some patients responded very well to the treatment [156].

An overview of the targets and inhibitors described here is given in Table 1.

## 5. Conclusions and Perspectives

Transcriptional dysregulation leading to the appropriation of an oncogenic gene expression program is an essential event responsible for the acquisition of cancer cell hallmarks such as proliferation, replicative immortality, and metastasis [76]. Indeed, oncogenic drivers are often regulators of transcription as exemplified by the AR and ERG in prostate cancer. Downstream effectors are frequently also transcriptional regulators, like for instance the Myc family members [202,203]. More recently, the essential role of BRD4 in multiple tumor types has been evidenced [127,128,204]. An increasing number of SNPs and small mutations are being found in non-coding regions of prostate tumors and are critical for the dysregulation of transcriptional programs in various ways. Finally, oncogenic, de novo acquired super-enhancers have recently been defined. They play critical roles in cancer as platforms for the recruitment of transcription factors and epigenetic players upstream of signaling pathways with an essential oncogenic role and are sensitive to perturbation [43,44,45,50,75,76]. As these mutations do not affect protein sequences, they will not be recognized by the immune system and therefore escape immune checkpoint therapies.

Targeting transcriptional regulators may often be more challenging in comparison to addressing proteins involved in signaling networks due to the absence of highly druggable pockets, multiple protein interactions, and nuclear localization. Concerning histone-modifying enzymes and readers, achieving sufficient selectivity is sometimes problematic, but ongoing efforts to identify chemical probes for the main epigenetic players represent a first important step [205]. This should however not deter endeavors that address early, master oncogenic drivers as this approach has the potential to better tackle tumor heterogeneity and may delay the emergence of resistance mutations, due to the multiple biological functions of these factors. On the other hand, the pleiotropic effects one might observe following blockade of such essential regulators may limit the therapeutic window so that a bespoke design of studies, for instance adaptive clinical trials, will possibly increase the chances of success [92,206,207].

## Figures and Tables

**Table 1:** Prostate cancer targets involved in gene regulation and selected inhibitors. Only the most advanced clinical studies are indicated.

Target	Target Function	Compound	Mode of Action	Status	Identifier
AR	Transcription factor	Enzalutamide Apalutamide Darolutamide	Competitive antagonists	FDA-approved FDA-approved Phase 3 active Phase 3 active	NCT00974311 NCT01212991 NCT02003924 NCT01946204 NCT02200614 NCT02799602
		ARV-110 ARD-69 ASC-J9	Degraders	Phase 1 recruiting Preclinical Preclinical	NCT03888612
		EPI-506	N-terminal domain binder	Phase 1/2 terminated	NCT02606123
		EZN-4176 AZD-5312	Antisense oligonucleotides	Phase 1a/1b suspended Phase 1 completed	NCT01337518 NCT02144051
AR/CYP 17A	Transcription factor/cytochrome	ODM-204	Dual inhibitor	Phase 2 ongoing	NCT02344017
ERG	Transcription factor	YK-4-279	Helicase interaction inhibitor	Preclinical	
		NSC139021	Ribosomal biogenesis regulator	Preclinical	
BET/BRD4	Acetylated lysine reader	GSK525762 ABBV-075 ABBV-744 GS-5829 ZEN003694 ZEN003694	Bromodomain inhibitors	Phase 1B ongoing Phase 1 active Phase 1 ongoing Phase 1 ongoing Phase 1 completed Phase 1 active	NCT03150056 NCT02391480 NCT03360006 NCT02607228 NCT02705469 NCT02711956
CDK7	Part of transcription factor II complex	THZ1	Kinase inhibitor	Preclinical	
CDK9	Part of P-TEFb complex	Atuveciclib MC180295	Kinase inhibitors	Phase 1 completed Preclinical	NCT02345382
CBP/p300	Transcriptional coactivator	CCS1477 GNE-049 32h	Bromodomain inhibitors	Phase 1/2 ongoing Preclinical Preclinical	NCT03568656
		A-485	Acetyl-transferase inhibitor	Preclinical	
EZH2	H3 lysine 27 methyl-transferase	CPI-1205 GSK126	Methyl-transferase inhibitors	Phase 1b/2 ongoing Preclinical	NCT03480646
EED	EZH2 interactor	MAK683	Inhibits H3K27me3 binding	Phase 1/2 ongoing	NCT02900651
Aurora A	Serine/threonine kinase	Alisertib	Kinase inhibitor	Phase 2 completed	NCT01799278
